# Long Noncoding RNA *H19* Inhibits Differentiation and Function of Secretory Lineage Cells in the Intestinal Epithelium by Targeting ATOH1

**DOI:** 10.3390/ncrna12050037

**Published:** 2026-09-15

**Authors:** Min S. Kwon, Hee K. Chung, Hongxia Chen, Amy VanderStoep, Bridgette Warner, Haonan Zhao, Prathik Atluri, Emnet Tesfayohannes, Douglas J. Turner, Lan Xiao, Jian-Ying Wang

**Affiliations:** 1 Cell Biology Group, Department of Surgery, University of Maryland School of Medicine, Room S243, HSF-II, 20 Penn St., Baltimore, MD 21201, USAprathik.atluri@gmail.com (P.A.);; 2 Baltimore Veterans Affairs Medical Center, 10 North Greene St, Baltimore, MD 21201, USA; 3 Department of Pathology, University of Maryland School of Medicine, Room S243, HSF-II, 20 Penn St., Baltimore, MD 21201, USA

**Keywords:** long noncoding RNAs, Paneth cells, goblet cells, mucosal maturation, gut barrier, transcription factors

## Abstract

**Background/Objectives**: Intestinal mucosal function relies on the integrated contribution of multiple cell lineages, including secretory cells. Long noncoding RNA *H19* modulates many cell processes essential for human pathologies. Here, we investigated the role of *H19* in development and function of secretory lineage cells and elucidated the implication of altered ATOH1 by *H19* in dysfunction of the secretory cells. **Methods**: Studies were conducted in *H19*-transgenic (H19-Tg) and *H19*-knockout (H19^−/−^) mice, intestinal organoids, and Caco-2 cells. Secretory lineage cells were examined by fluorescent immunostaining with their specific cell markers, including Lysozyme, Mucin2, DCLK1, and Chromogranin A. Intestinal mucosal growth was measured by BrdU incorporation, and intestinal permeability was detected by paracellular tracer flux assay using FITC-Dextran. Single cell RNA-sequence, quantitative real-time PCR, and Western immunoblotting analyses were used to monitor changes in gene expression. **Results**: Tissue-specific transgenic expression of *H19* in the intestinal epithelium caused defects in Paneth, goblet, tuft, and enteroendocrine cells in mice, whereas targeted deletion of the *H19* gene increased the function of secretory lineage cells. Locally increasing the levels of *H19* in the epithelium impaired gut barrier function in H19-Tg mice, although it failed to alter mucosal growth. The intestinal mucosal tissues of H19-Tg mice exhibited a remarkable decrease in the levels of differentiation-associated transcription factors including ATOH1. Ectopically expressed ATOH1 in the *H19*-enriched intestinal organoids derived from H19-Tg mice rescued development of the secretory lineage cells. Mechanistically, *H19* inhibited ATOH1 expression via inhibition of its translation. **Conclusions**: These findings indicate that *H19* functions as a negative regulator of differentiation and function of secretory lineage cells in the intestinal mucosa at least partially via the inhibition of ATOH1 translation.

## 1. Introduction

The mammalian small intestinal epithelium facilitates selective uptake of nutrients and functions as a physical barrier protecting the subepithelial tissue against luminal noxious substances and microbial pathogens. The epithelium undergoes a rapid and continual self-renewal that is primarily driven by intestinal stem cells (ISCs) located at the bottom of crypts [1,2]. The intestinal epithelium comprises different cell lineages that can be divided into two broad categories: absorptive cells and secretory cells [3,4,5]. Enterocytes account for the majority (~80%) of all differentiated cells and are efficient nutrient- and water-absorbing epithelial cells. Secretory cells include Paneth cells that produce antibacterial compounds and niche factors for ISCs, mucus-secreting goblet cells, chemosensory tuft cells, and hormone-producing enteroendocrine cells; all these lineages are essential for the gut epithelium-host defense, constant epithelial renewal, mucosal immunity, and gut barrier function [3,6,7]. Rapid proliferation of ISCs in the crypts generates transit-amplifying progenitor cells that divide two to five times before maturing into enterocytes, while secretory cells may differentiate directly from a stem cell [8,9]. Differentiation of the secretory lineage cells in the intestine is tightly controlled by numerous transcription factors including the key transcription factor ATOH1 in prosecretory cells, its repressor HES1, and other transcription factors such as SOX9, SPDEF, POU2F3 and neuroG3 that are crucial for the maturation of specific secretory lineages [3,10]. Dysfunction of secretory lineage cells in the intestinal mucosa occurs commonly in various pathologies, leading to defects in the epithelial integrity and gut barrier function [11,12,13].

The majority of the mammalian genome (>96%) is transcribed into a large array of noncoding RNAs (ncRNAs) that have emerged as important players and regulators in diverse cellular processes and human diseases [14,15]. Conversely, protein-coding sequences are a minority (<4%) of the transcriptional output [16]. Long ncRNAs (lncRNAs) are defined as transcripts spanning >200 nucleotides in length and are expressed in cell type- and differentiation stage-dependent manners in various tissues and organs [17,18]. LncRNAs function as molecular scaffolds, decoys, or signals to regulate virtually every level of gene expression and cellular processes, and their deregulation is intimately involved in diverse human disorders [15,16,17,18]. LncRNAs work jointly with microRNAs (miRNAs), RNA-binding proteins (RBPs), and other gene regulators to govern gene transcription, mRNA stability or/and translation [16,17,18,19]. Our previous work has revealed several gut mucosal enriched lncRNAs, including *SPRY4-IT1*, *uc.173*, *uc.230*, and *GAS5*, which are shown to play an important role in maintaining the epithelial homeostasis via control of constant mucosal renewal, wound healing, and gut permeability in stressful environments [20,21,22,23]. For instance, increased levels of *SPRY4-IT1* strengthens the intestinal barrier function via interacting with RBP HuR [23], while *uc.173* stimulates regeneration of the intestinal mucosa and enhances gut barrier function by modulating mitochondrial metabolism and tight junctions’ expression [23,24,25]. *Uc.230* protects the intestinal epithelium against apoptosis and promotes injury-induced epithelial regeneration by regulating CUG-binding protein 1 (CUGBP1) via sequestering miR-503 [26]. Recently, increased *GAS5* was found to induce enteric neuroplasticity through its interplay with the miR-23/NMDA NR2b axis [27] and to disrupt the gut barrier function by stimulating the expression of small noncoding vault RNAs [28].

LncRNA *H19* is transcribed from the conserved imprinted *H19*/*Igf2* gene cluster located on human chromosome 11p5.5 and affects many cellular processes central to human pathologies [29,30]. During embryogenesis, *H19* is highly expressed in most tissues, but its levels decline dramatically after birth [30,31]. Increased *H19* represses embryonic placental growth and alters the expression of imprinted genes during fetal development. Targeted deletion of the mouse *H19* locus results in an overgrowth phenotype with an increase in body weight, which is abolished by the transgenic re-expression of the *H19* gene [30]. The increased expression of *H19* is commonly observed in a broad spectrum of pathophysiological conditions, such as various cancers [31,32], exposure to hypoxia or estrogens [33,34], sepsis [35], and the inflamed intestinal mucosa [35,36]. However, there has not been any report on major shifts in the canonical genomic imprinting status of *H19* in non-malignant conditions where the intestinal epithelial integrity is damaged. Emerging evidence indicates that *H19* regulates distinct cellular processes and is implicated in various human diseases by either acting as primary miRNA template for miR-675 [37], working as a molecular sponge for target miRNAs including let-7 and miR-145-5p [36,38], or interacting with RBPs.

Our previous study shows that ectopically expressed *H19* in cultured intestinal epithelial cells (IECs) disrupts the epithelial barrier function in an in vitro model via release of miR-675 embedded in *H19* exon 1 [21]. In an in vivo study, we demonstrate that ablating *H19* gene in mice protects the intestinal barrier against septic stress by enhancing function of Paneth and goblet cells and promoting autophagy [35]. Here, we investigated the role of *H19* in the regulation of development and function of secretory lineage cells in the small intestinal epithelium and identified *H19* as an inhibitor of secretory cell differentiation by inhibiting ATOH1 translation.

## 2. Results

### 2.1. H19 Deletion Alters Cell Type-Specific Gene Expression Profiles in the Intestinal Mucosa

**Figure 1:**
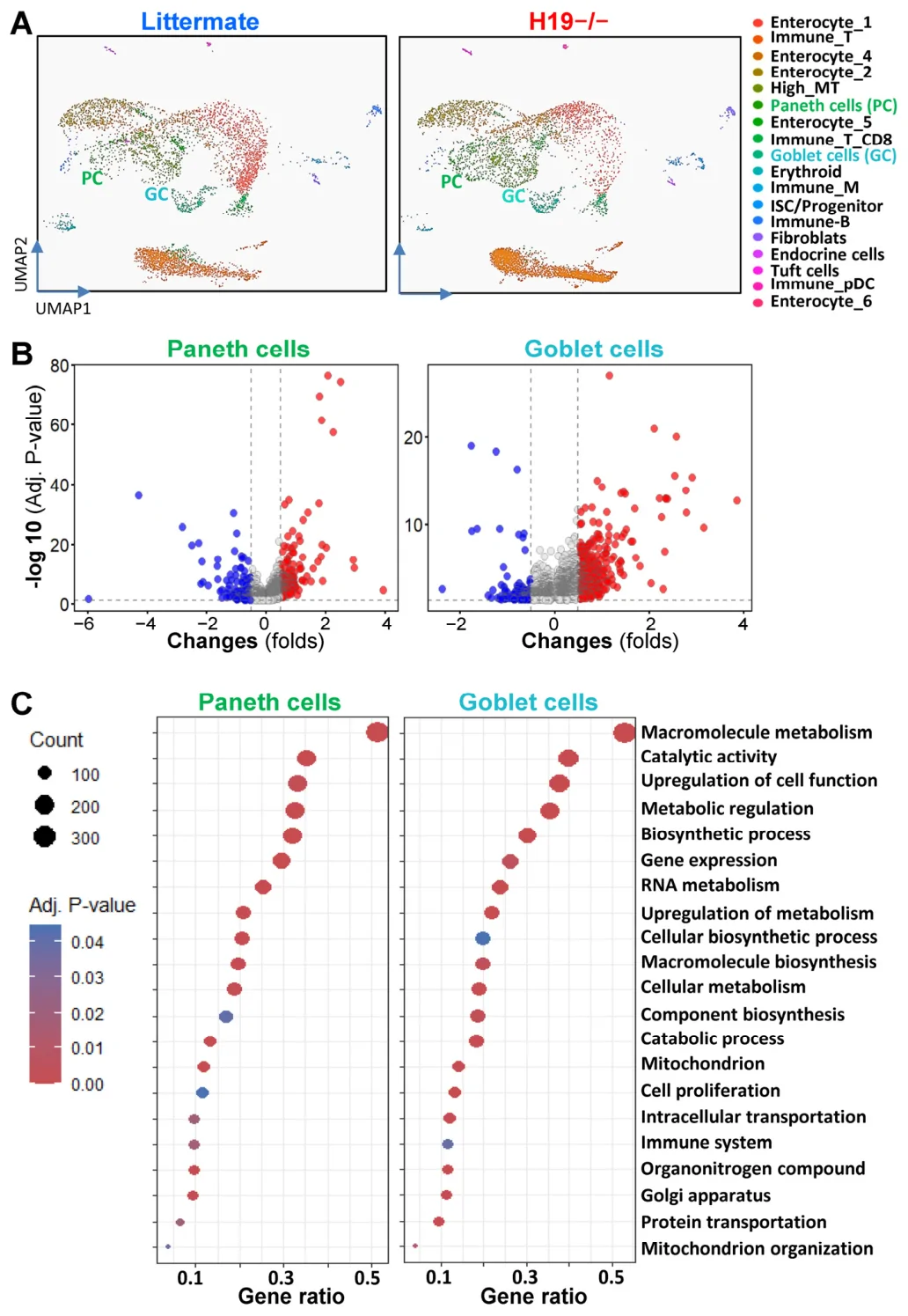
Single-cell transcriptional profiles of Paneth cells and goblet cells in the small intestinal epithelium after deletion of the *H19* in mice. (**A**) Uniform manifold approximation and projection of key epithelial cell types of the small intestinal mucosa from control littermate (**left**) and H19^−/−^ mice (**right**). Annotated clusters of Paneth cells are shown in green; goblet cells are shown in light-green. (**B**) Scatter plot depictions of genes expressed differentially in Paneth cells (**left**) and goblet cells (**right**) in the small intestinal mucosa of littermate and H19^−/−^ mice, as measured by scRNA-seq analysis. Values are the means from two separate experiments. (**C**) Causal analysis of most altered pathways involved in gene transcription and various cellular processes in Paneth cells (**left**) and goblet cells (**right**) after *H19* deletion in mice.

To investigate the role of *H19* in the intestinal epithelial cell-specific transcriptome and cell lineage, single-cell RNA-sequencing (scRNA-seq) analysis was performed on the fresh tissues of small intestinal mucosa harvested from *H19*^∆Exon1^ (*H19*^−/−^) mice carrying a maternal deletion of exon 1 in the *H19* gene [35,39] and control littermates. About 40,000 live cells were captured, as quality characterized and validated by flow-cytometry analysis as described previously [12,23], and grouped into 18 clusters that expressed classic cell type markers for the lineages of absorptive and secretory cells, aligned with published single-cell transcriptomic signatures [40]. Specifically, the secretory cells in the epithelial compartment of the small intestine consisted of goblet cells, tuft cells, Paneth cells, and enteroendocrine cells in both groups, while the absorptive cells included various enterocytes (Figure 1A). Of note, goblet cells expressed high levels of transcripts including *Myo5c*, *Atoh1*, *spink4*, *Muc2Zg16*, and *Tff3*, and Paneth cells had marked expression of *Reg3a* and *Defa5* mRNAs, similar to others reports [12,40,41]. Enteroendocrine cells were marked by *Chgb*, *Chga*, *Cck*, *Sct*, *Ghrl*, *Pyy*, and *Ambp*; tuft cells were marked by *Trpm5*, *Dclk1*, *Sox9*, *Sucnr1*, *Sh2d6*, and *Rgs13*, while ISCs were marked by transcripts including *Olfm4*, *Rgmb*, *Ascl2*, *Lgr5*, and *Smoc2* mRNAs. Cell cluster analysis suggested that the numbers of goblet cells and Paneth cells were increased in H19^−/−^ mice compared to littermates, which were consistent with the findings obtained from studies using immunostaining assays in our previous work [35]. However, there was no significant difference in the numbers of enteroendocrine cells, tuft cells, and ISCs in the small intestinal mucosa between H19^−/−^ mice and control littermates.

The cell type-specific gene expression profiling demonstrated the existence of transcriptionally distinct Paneth cells and goblet cells in the small intestinal epithelium of H19^−/−^ mice. We compared the transcriptomic profiles from H19^−/−^ mice to control littermates and found that ~348 RNAs were differentially expressed in the Paneth cells, with 128 genes less abundant and 220 genes more abundant after *H19* deletion (Figure 1B, left). In goblet cells, ~480 RNAs were differentially expressed, 128 RNAs were lower and 352 RNAs were higher in H19^−/−^ mice compared to the mucosa of control littermates (Figure 1B, right). Causal analysis to screen for function-associated gene expressions in our single-cell sequencing data originated from Paneth cells and goblet cells further suggested that targeted deletion of the *H19* gene in mice increased the activity of Paneth cells and goblet cells, as supported by significantly increased expression of mRNAs encoding proteins that are in charge of essential cellular processes and metabolism (Figure 1C). These activated pathways in the *H19*-deficient epithelium included the processes involved in macromolecular metabolism, catalytic activity, cellular biosynthesis, gene expression, RNA metabolism, and mitochondrial structure and function. Among these differentially abundant mRNAs are also many encoding transcription factors and regulatory proteins that are necessary for differentiation and function of secretory lineage cells in the intestinal epithelium. Since these cellular processes and transcriptional factors are essential for the function and effectiveness of Paneth cells and goblet cells [3,4], their activation by decreasing the levels of cellular *H19* is implicated in maintaining the intestinal epithelium homeostasis in stressful environments. In summary, the data from our single-cell RNA sequencing clearly pointed out transcriptomic and cellular responses to *H19* deletion in the small intestinal mucosa, and suggested an essential role of *H19* in regulating Paneth and goblet cells in the intestinal epithelium.

### 2.2. Transgenic Expression of H19 in Mice Results in Defects in Secretory Lineage Cells

**Figure 2:**
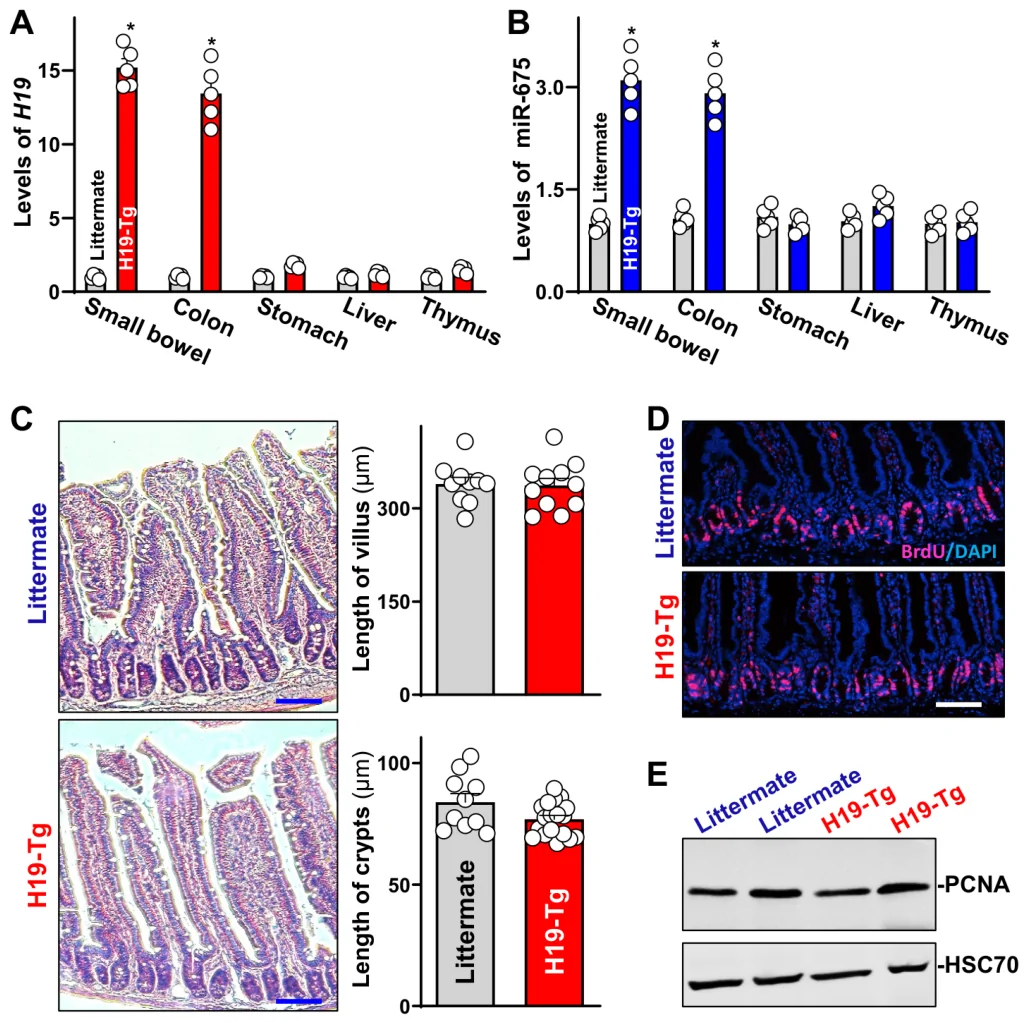
Characterization of intestinal epithelium-specific *H19* transgenic (H19-Tg) mice. (**A**) Levels of tissue *H19* in the small intestinal mucosa, colon, stomach, liver, and thymus as measured by RT-qPCR analysis. Total RNA was isolated and used in RT-qPCR reactions, and equal loading was monitored by assessing *Gapdh* mRNA levels. Values are the means ± SEM (*n* = 5). * *p* < 0.05 compared with control littermate mice. (**B**) Levels of mucosal miR-675 in various tissues as described in (**A**). Values are the means ± SEM (*n* = 5). * *p* < 0.05 compared with littermates. (**C**) Photomicrographs of hematoxylin and eosin (**left**) and changes in the length of villi and crypts (**right**) of the intestinal mucosa described in (**A**) (*n* = 10). (**D**) Proliferating cells in small intestinal crypts in control littermate and H19-Tg mice as measured by BrdU immunostaining. Red, Ki67; Blue, DAPI. Scale bars: 50 μm. (**E**) Immunoblots of growth-associated protein PCNA in the small intestinal mucosa. Whole-cell lysates were harvested from littermate and H19-Tg mice and prepared for Western immunoblotting analysis. HSC70 immunoblotting was performed as an internal control for equal loading. In (**A**–**C**), statistical significance was analyzed using unpaired, two-tailed Student’s *t* test. In (**D**,**E**), experiments were repeated three times and showed similar results.

The basal level of *H19* in the intestinal mucosa is relatively low [21,35]. To further investigate the role of *H19* in the maturation of secretory lineage cells in vivo, we created intestinal epithelial tissue-specific *H19* transgenic (H19-Tg) mice by using the A33 promoter, as described previously [42]. Age-matched H19-Tg mice and littermate controls (6–9 weeks old, both male and female) were used for phenotype comparison. H19-Tg mice exhibited a specific overexpression of *H19* in the mucosal tissues of the small intestine and colon, but not in gastric mucosa, liver, and thymus (Figure 2A), as measured by quantitative real-time PCR (qRCR) analysis [43,44]. The levels of mucosal *H19* in H19-Tg mice increased by ~15-fold in the small intestine and ~13-fold in colon, respectively, when compared with those observed in control littermates. Transgenic overexpression of *H19* also specifically induced the expression levels of miRNA-675 (Figure 2B) that is embedded in *H19* exon 1. However, overexpression of *H19* did not change the levels of gene expression of other miRNAs such as miR-29b and miR-222. H19-Tg mice were generally normal and healthy, with no significant differences in appearance, body weight, gastrointestinal gross morphology, or reproductivity compared to control littermates.

*H19* transgenic expression in the intestinal epithelium did not impact the renewal of the intestinal mucosa in vivo. There were no significant alterations in histological features of the small intestinal mucosa (Figure 2C, left), the lengths of villi and crypts (Figure 2C, right), the proliferating crypt cell population marked by BrdU incorporation (Figure 2D), and the levels of proliferation marker PCNA (Figure 2E) between H19-Tg mice and control littermates. In addition, *H19* overexpression did not directly induce apoptosis in the intestinal epithelium, since there was no significant increase in cell death observed in H19-Tg mice, as examined by TUNEL staining [42]. These results indicate that H19-Tg mouse is an excellent tissue-specific gain-of-function model of *H19* for studying maturation and function of the secretory lineage cells in the intestinal epithelium.

**Figure 3:**
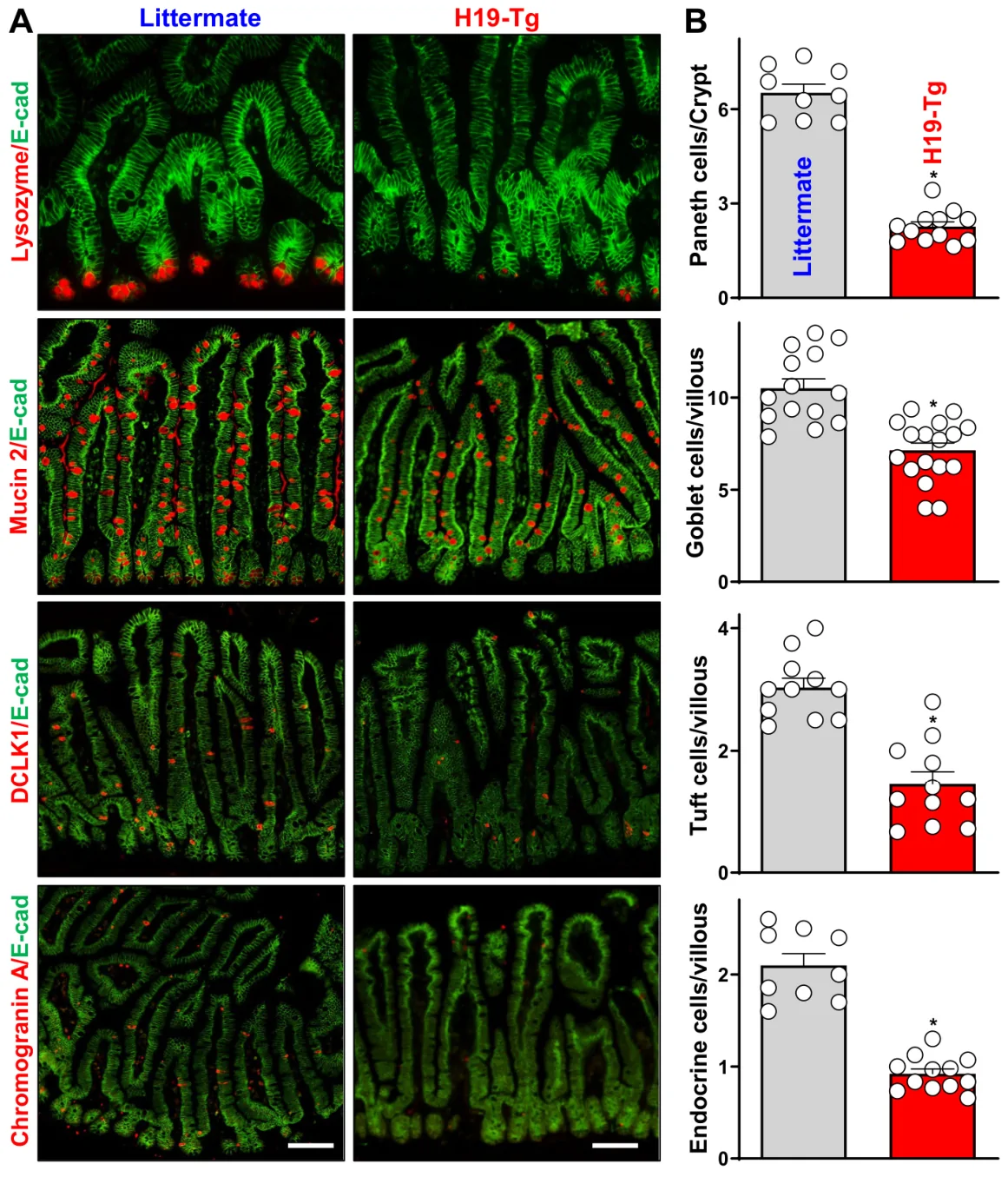
Transgenic expression of *H19* in mice inhibits development of secretory lineage cells in the small intestinal epithelium. (**A**) Immunostaining of Paneth (lysozyme-positive), goblet (mucin 2-positive), tuft (DCLK1-positive), and enteroendocrine (chromogranin A-positive) cells in the small intestinal mucosa of littermate and H19-Tg mice. Red, lysozyme, mucin 2, DCLK1 or chromogranin A; and green, E-cadherin (E-cad). Scale bars: 50 μm. (**B**) Summarized results from studies described in (**A**). Values are the means ± SEM (*n* = 10). * *p* < 0.05 compared with control littermate mice. In (**B**), statistical significance was analyzed using unpaired, two-tailed Student’s *t* test.

**Figure 4:**
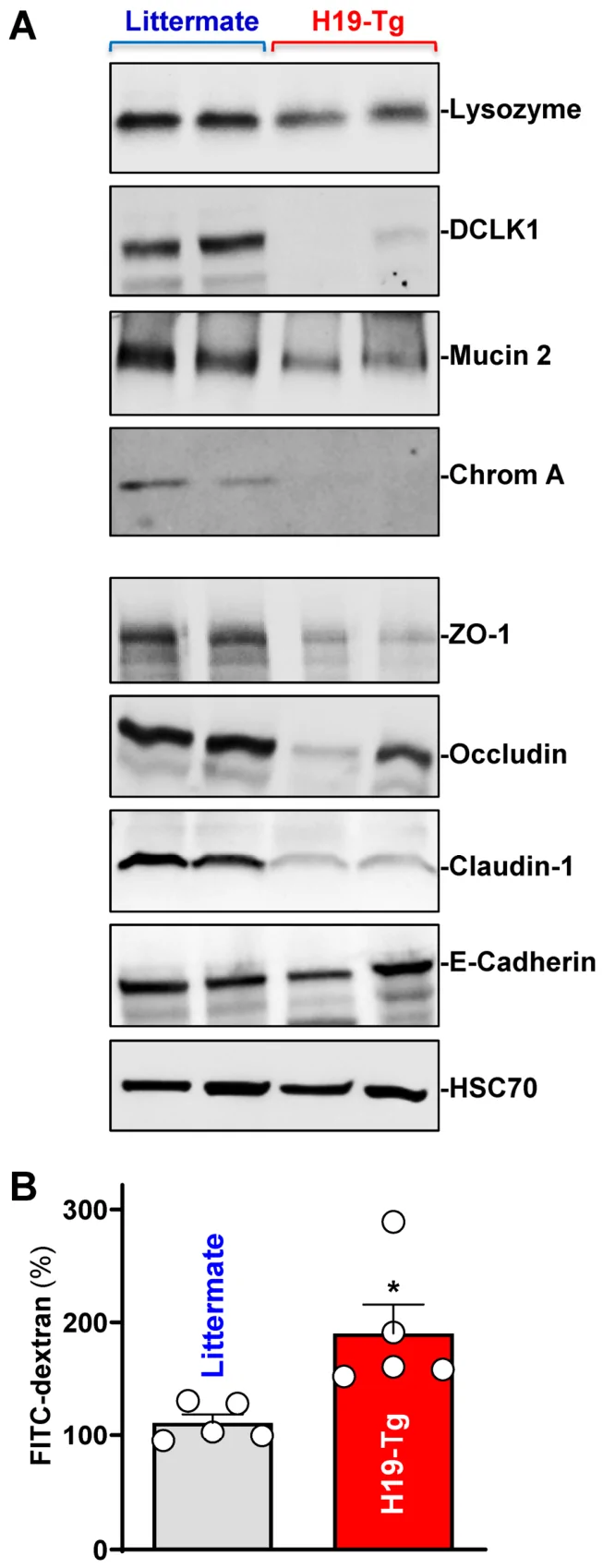
Transgenic *H19* expression decreases the levels of marker proteins of secretory lineage cells and reduces the abundance of tight junction proteins in the small intestinal mucosa. (**A**) Immunoblots of lysozyme, mucin 2, DCLK1, chromogranin A (Chrom A), and tight junction proteins in the small intestinal mucosa of littermate and H19-Tg mice. HSC70 immunoblotting was performed as an internal control for equal loading. (**B**) Gut permeability in mice described in (**A**). FITC dextran was given orally, and blood samples were collected 4 h thereafter for measurement. Values are the means ± SEM (*n* = 5). * *p* < 0.05 compared with control littermates. In (**B**), unpaired, two-tailed Student’s *t* test was used for statistical analysis.

Tissue-specific transgenic expression of *H19* in the intestinal epithelium caused defects in secretory lineage cells, such as Paneth cells, goblet cells, tuft cells, and enteroendocrine cells in the small intestinal mucosa of mice, as indicated by immunofluorescence staining [11,35]. The tissue staining of the small intestine suggested that in control littermates, lysozyme-positive cells (Paneth cells) were located at the base of the intestinal crypts, but mucin 2-positive cells (goblet cells), double cortin-like kinase 1 (DCLK1)-positive cells (tuft cells), and chromogranin A-positive cells (enteroendocrine cells) were distributed at both villous and crypt regions (Figure 3A, left). The numbers of Paneth cells, goblet cells, tuft cells, and enteroendocrine cells in the small intestinal mucosa were markedly reduced in H19-Tg mice compared to control littermates (Figure 3A, right). Quantification of this reduction indicated that the numbers of intestinal Paneth cells, goblet cells, tuft cells and enteroendocrine cells in H19-Tg mice decreased by ~65%, ~33%, ~52%, and ~56%, respectively, compared with control littermate mice (Figure 3B). Moreover, the levels of marker proteins for each secretory cells such as lysozyme, mucin 2, DCLK1 and chromogranin A in the small intestinal mucosa also decreased in H19-Tg mice (Figure 4A, top), as measured by immunoblotting analysis [43]. However, transgenic *H19* overexpression failed to alter enterocyte differentiation in the small intestinal mucosa, as examined by villin immunostaining analysis.

As expected, transgenic *H19* overexpression in the intestinal epithelium disrupted the epithelial barrier function, as evidenced by decreased levels of tight junction proteins ZO-1, occludin, and claudin-1 in H19-Tg mice relative to control littermates (Figure 4A, bottom). Consistent with its effect on tight junction expression, H19-Tg mice exhibited increased basal levels of gut permeability compared to that observed in control littermates (Figure 4B), as measured by FITC-conjugated dextran assays [42]. On the other hand, increased *H19* did not affect the level of adherens junction protein E-cadherin in the small intestinal mucosa. Another line of H19-Tg mice generated from an independent founder was also examined and displayed similar defects in secretory lineage cells and gut barrier dysfunction.

### 2.3. H19 Decreases Levels of Transcriptional Factors Necessary for Secretory Cell Differentiation

**Figure 5:**
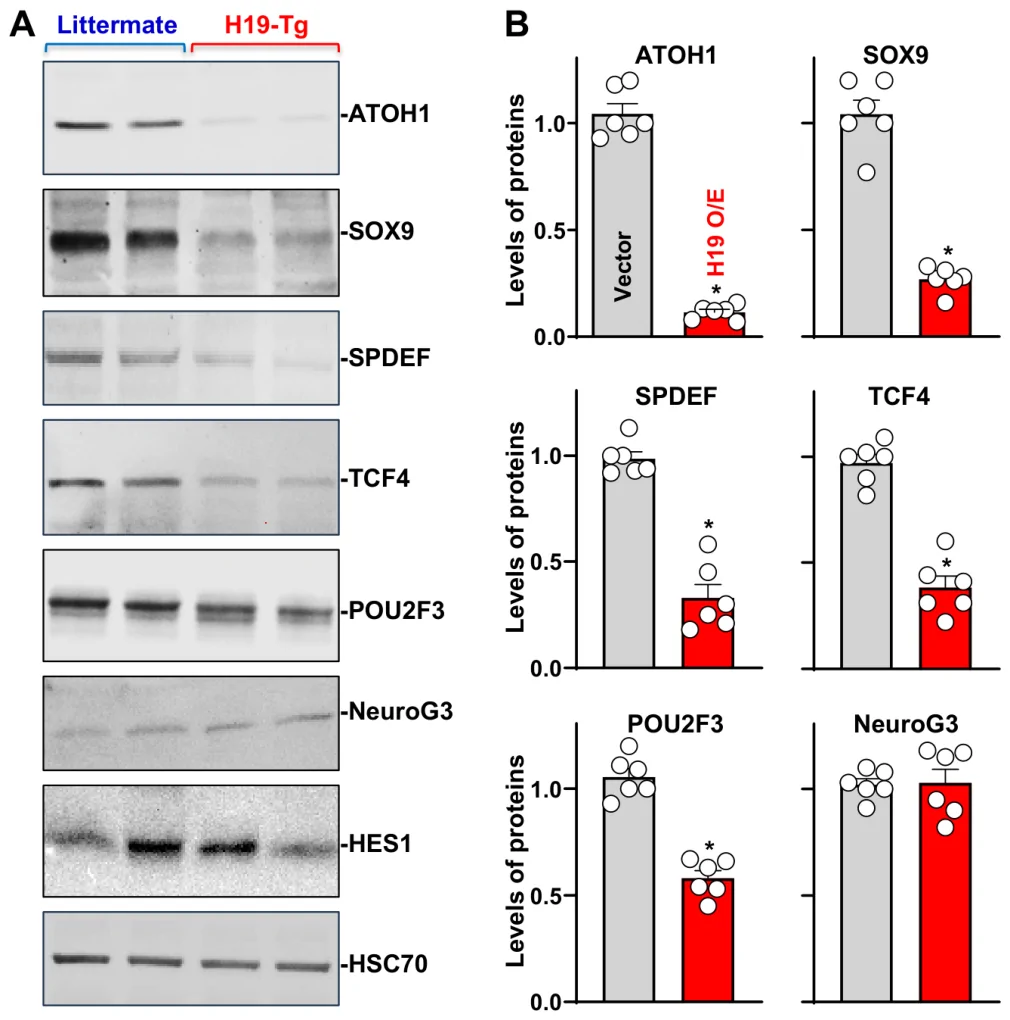
Transgenic *H19* expression decreases the levels of ATOH1 and other differentiation-associated transcription factors in the small intestinal mucosa. (**A**) Immunoblots of ATOH1, SOX9, SPDEF, Tcf4, POU2F3, NeuroG3, and HES1 in the small intestinal mucosa of littermate and H19-Tg mice. HSC70 immunoblotting was performed as an internal control for equal loading. (**B**) Summarized data from studies as described in (**A**). Values are the means ± SEM (*n* = 6). * *p* < 0.05 compared with control littermates. In (**B**), statistical significance was analyzed using unpaired, two-tailed Student’s *t* test.

To investigate the mechanism underlying *H19* in the regulation of secretory lineage cell maturation, we examined the expression levels of various transcription factors that are necessary for the differentiation of secretory lineage cells in the small intestinal epithelium. As shown in Figure 5 (top), the intestinal mucosa of H19-Tg mice exhibited a dramatical decrease in the levels of ATOH1, SOX9, SPDEF, TCF4, and POU2F3 proteins when compared with those observed in control littermates. Quantification analysis showed that the levels of tissue ATOH1, SOX9, SPDEF, TCF4, and POU2F3 proteins in the intestinal mucosa of H19-Tg mice decreased by ~88%, ~75%, ~68%, ~60%, and ~45%, respectively (Figure 5B). In contrast, there were no significant differences in the mucosal abundances of NeuroG3 and HES1 proteins in the small intestine between H19-Tg mice and control littermates (Figure 5A, bottom). These results indicate that locally increasing the levels of *H19* in the intestinal epithelium decreases the expression levels of several differentiation-associated transcription factors, including ATOH1, along with defects in secretory lineage cells.

### 2.4. Increased ATOH1 Rescues Maturation of Secretory Cells in H19-Enriched Intestinal Organoids

**Figure 6:**
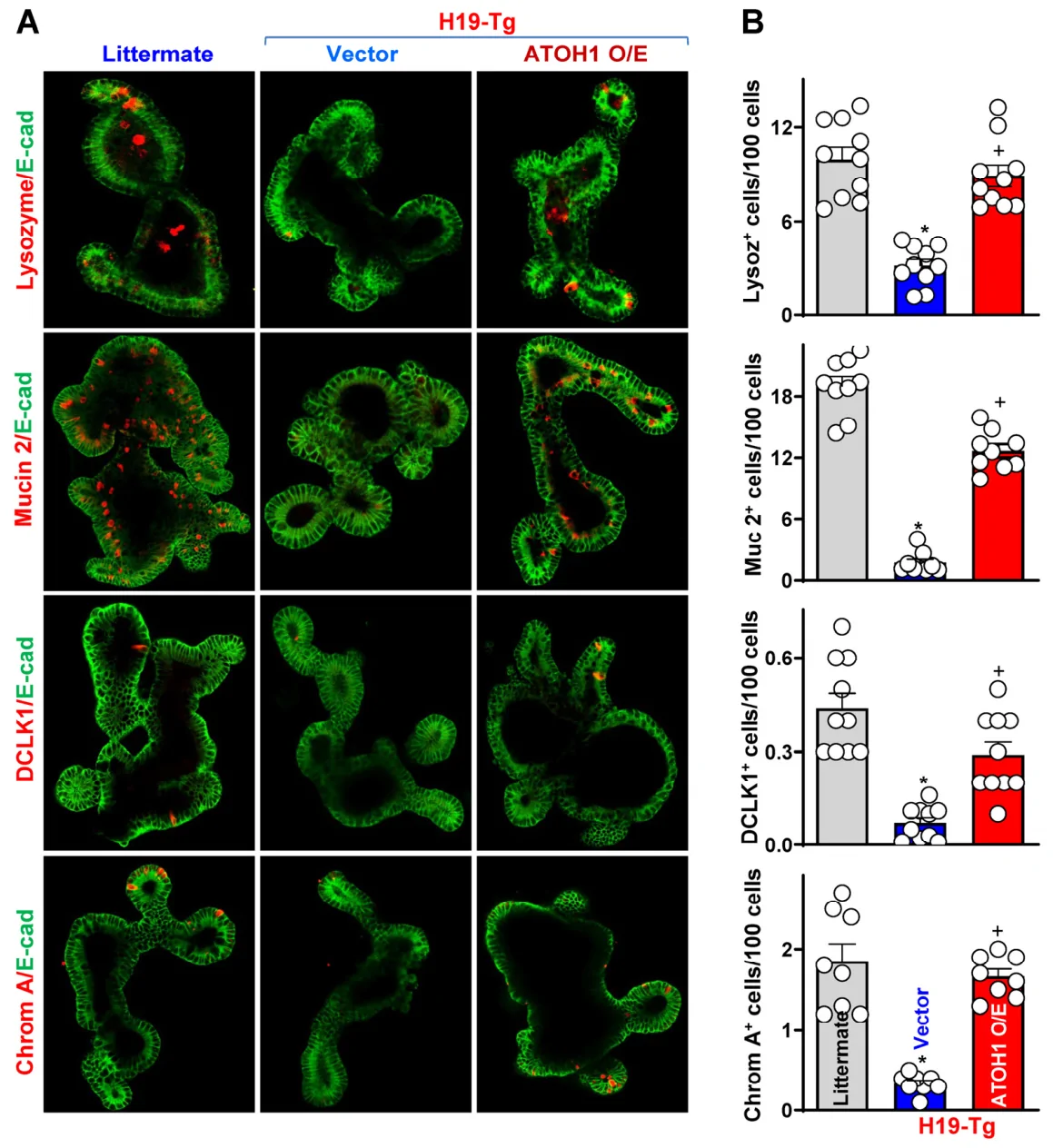
Ectopic overexpression of ATOH1 restores differentiation of secretory lineage cells in intestinal organoids derived from H19-Tg mice. (**A**) Immunostainings of Paneth cells (lysozyme-positive cells), goblet cells (mucin 2-positive cells), tuft cells (DCLK1-positive cells), and endocrine cells (chrom A-positive cells) in intestinal organoids 48 h after transfection with an ATOH1 expression vector or a control empty vector. (**B**) Quantitative data of these secretory lineage cells in intestinal organoids described in panel (**A**). Values are the means ± SEM (*n* = 10). * *p* < 0.05 compared with control littermates; ^+^ *p* < 0.05 compared with empty vector-transfected organoids derived from H19-Tg mice. In (**B**), statistical significance was analyzed using one-way ANOVA with Tukey’s post hoc test.

**Figure 7:**
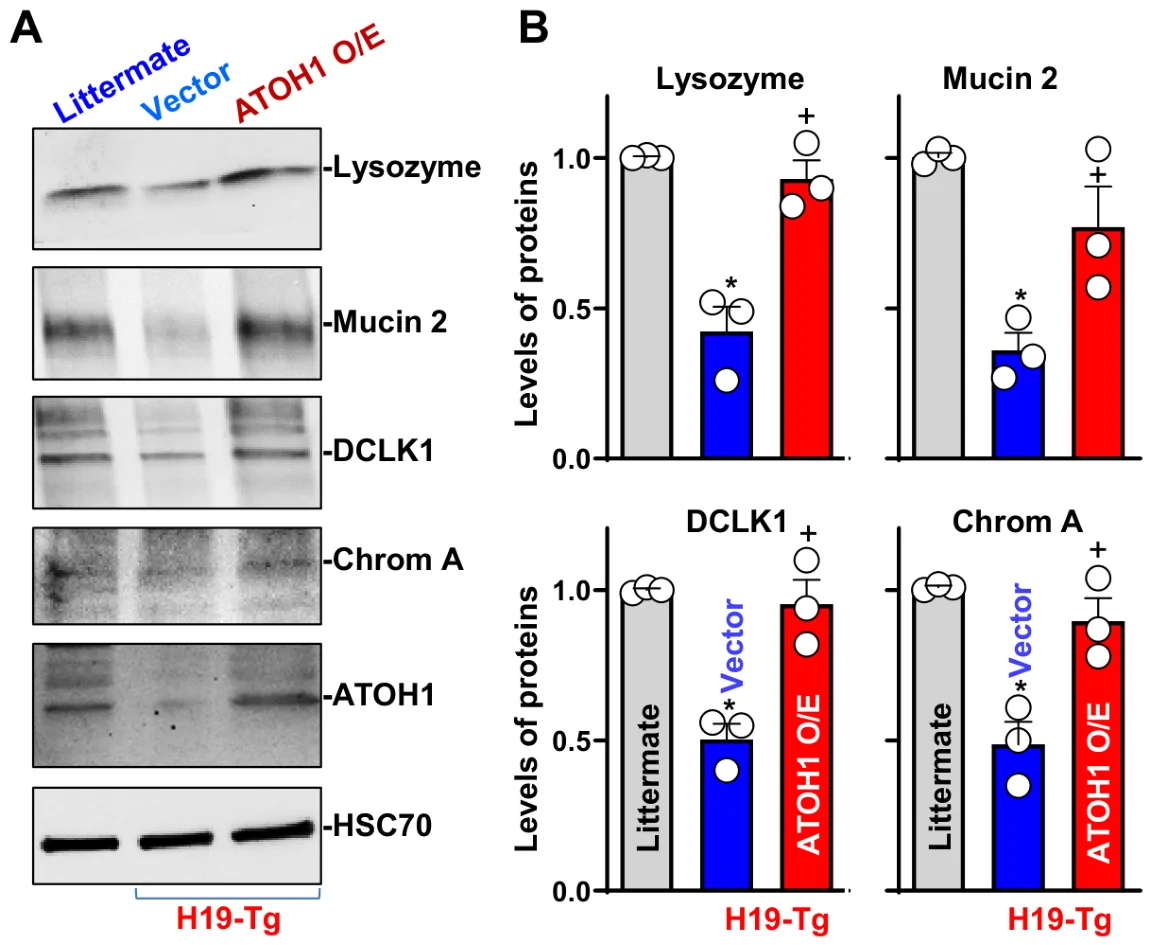
Ectopic overexpression of ATOH1 prevents a decrease in the levels of marker proteins of different secretory cells in intestinal organoids derived from H19-Tg mice. (**A**) Immunoblots of lysozyme, mucin 2, DCLK1 and chromogranin A (chrom A) proteins in intestinal organoids 48 h after transfection with control or ATOH1 expression vector. HSC70 immunoblotting was performed as an internal control for equal loading. (**B**) Summarized data from studies as described in (**A**). Values are the means ± SEM (*n* = 3). *^,+^ *p* < 0.05 compared with littermates and H19-overexpressed organoids transfected with control vector, respectively. In (**B**), statistical significance was analyzed using one-way ANOVA with Tukey’s post hoc test.

Since ATOH1 is the key prosecretory basic helix-loop-helix transcription factor and its loss leads to depletion of the secretory lineages in the intestinal epithelium [1,3], we elucidated the role of altered ATOH1 in *H19*-mediated defects in the secretory lineage cells in an ex vivo model using primarily cultured intestinal organoids. As described previously [35,45], intestinal organoids were derived from proliferating crypts of the small intestinal mucosa of H19-Tg and control littermate mice. By 5 days after initial culture, the structures of intestinal organoids from both H19-Tg and littermate mice consisted of multiple buds and cells. As expected, there were no significant differences in the growth of intestinal organoids between the two groups, as examined by the organoid surface area and BrdU incorporation. In agreement with the observations in vivo, intestinal organoids derived from H19-Tg mice exhibited decreased numbers of secretory lineage cells, including Paneth cells, goblet cells, tuft cells and enteroendocrine cells, when compared with those observed in the organoids generated from littermate mice (Figure 6A, middle). Quantification of these results showed that the numbers of Paneth cells, goblet cells, tuft cells, and enteroendocrine cells in the organoids derived from H19-Tg mice decreased by ~68%, ~90%, ~84%, and ~82%, respectively, compared to those observed in the organoids from littermate mice (Figure 6B, middle). Consistently, the levels of lysozyme, mucin 2, DCLK1, chromogranin A, and ATOH1 proteins in the intestinal organoids derived from H19-Tg mice were also decreased (Figure 7), as measured by immunoblotting analysis. These results indicate that defects in secretory lineage cells in H19-Tg mice results primarily from a cell-autonomous regulatory effect of this lncRNA but not from the effect of secreted factors in the gut.

To examine the effect of increasing the levels of ATOH1 on differentiation of the secretory lineage cells in intestinal organoids isolated from H19-Tg mice, we constructed a plasmid vector expressing ATOH1 under control of the pCMV promoter as reported previously [46]. Ectopically expressed ATOH1 in the *H19*-enriched organoids derived from H19-Tg mice improved development of the secretory lineage cells, as evidenced by a significant increase in the numbers of Paneth cells, goblet cells, tuft cells, and enteroendocrine cells in the organoids transfected with the ATOH1 expression vector relative to those transfected with the empty control vector (Figure 6A,B, right). Consistently, the levels of lysozyme, mucin 2, DCLK1, and chromogranin A proteins were also returned to near normal levels in the organoids derived from H19-Tg mice after ATOH1 overexpression (Figure 7). These data indicate that defects in development and differentiation of secretory lineage cells in the intestinal organoids derived from H19-Tg mice result primarily from the inhibition of ATOH1.

### 2.5. H19 Inhibits ATOH1 Expression via Repression of Its Translation

**Figure 8:**
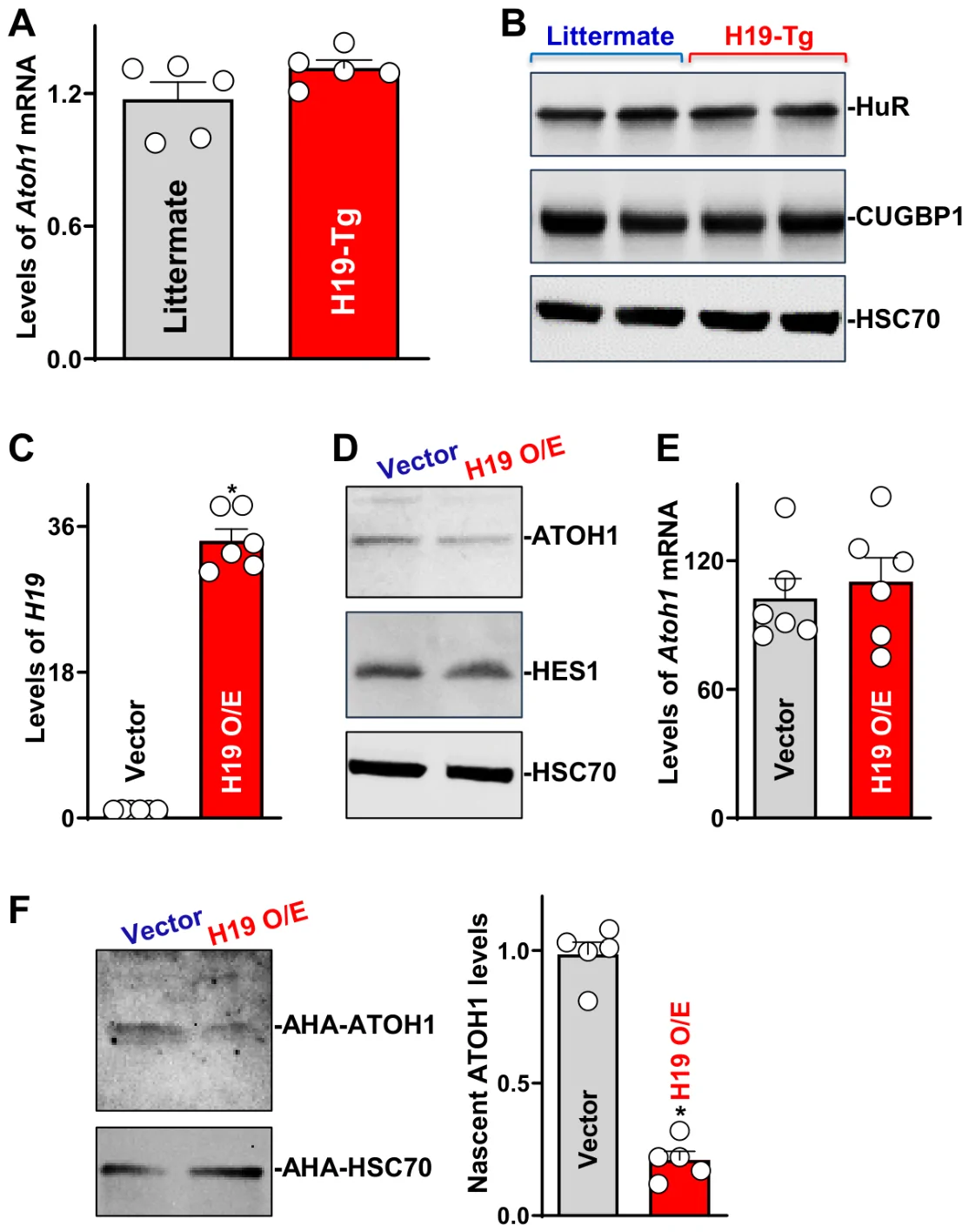
Ectopic H19 overexpression inhibits translation of ATOH1 in cultured IECs. (**A**) Levels of the *Atoh1* mRNA in the small intestinal mucosa of littermate and H19-Tg mice. Total RNAs were isolated from the tissue and examined by RT-qPCR analysis. Values are the means ± SEM (*n* = 5). (**B**) Immunoblots of RNA-binding proteins HuR and CUGBP1 in the mucosal tissue described in (**A**). HSC70 immunoblotting was performed as an internal control for equal loading. (**C**) Levels of cellular *H19* in Caco-2 cells 48 h after transfection with the *H19* expression vector. Values are the means ± SEM (*n* = 6). * *p* < 0.05 compared with control vector. (**D**) Immunoblots of ATOH1 and HES1 proteins in cells described in (**C**). (**E**) Levels of the *Atoh1* mRNA in cells described in (**C**) (*n* = 6). (**F**) Newly synthesized ATOH1 protein as measured by L-azidohomoalaine (AHA) incorporation assays in cells described in (**C**). Values are the means ± SEM (*n* = 5). * *p* < 0.05 compared with control vector. In (**A**,**C**,**E**,**F**), statistically significant was analyzed using unpaired, two-tailed Student’s *t* test. In (**B**,**D**), experiments were repeated three times and showed similar results.

To examine the mechanism by which *H19* regulates ATOH1 expression, we examined levels of the *Atoh1* mRNA in the intestinal mucosa and demonstrated that increasing the levels of *H19* did not alter transcription of the *Atoh1* gene, since there were no significant differences in the levels of *Atoh1* mRNA between H19-Tg mice and control littermates (Figure 8A). To determine the involvement of RBPs in this process, we examined changes in the levels of mucosal HuR and CUG-binding protein 1 (CUGBP1) in the small intestine of H19-Tg mice. Our results revealed that transgenic expression of *H19* failed to affect expression levels of RBPs, since the levels of tissue HuR and CUGBP1 in the intestinal mucosa of H19-Tg mice were indistinguishable from those observed in control littermate mice (Figure 8B).

In cultured IECs, ectopically expressed *H19*, by transfection with its expression vector (Figure 8C), decreased the levels of ATOH1 protein (Figure 8D), although it did not alter level of the *Atoh1* mRNA (Figure 8E). Consistent with the observation in H19-Tg mice, *H19* overexpression did not reduce the abundance of cellular HES1 protein in IECs. However, ectopically expressed *H19* inhibits ATOH1 translation, since the levels of newly synthesized ATOH1 protein decreased dramatically in cells overexpressing *H19* relative to cells transfected with control vector (Figure 8F). The translational rate of ATOH1 in *H19*-transfected cells decreased by ~79%, compared with those from cells transfected with control vector. These results strongly suggest that *H19* inhibits ATOH1 expression primarily by decreasing *Atoh1* mRNA translation in the intestinal epithelium.

## 3. Discussion

In response to different stresses, activation and effectiveness of secretory lineage cells in the intestine are essential for sustaining the epithelial homeostasis by enhancing the epithelium-host defense, constant mucosal renewal, immune-response, and gut barrier function [3,4,10]. Our previous study showed that targeted deletion of *H19* in mice increases the function of Paneth and goblet cells, thus promoting gut barrier function [35], but the exact mechanism underlying the control of secretory lineage-cell function by *H19* remains unknown. The present study provides genetic evidence demonstrating the importance of *H19* in regulating the development and maturation of secretory lineage cells via the posttranscriptional control of transcription factor ATOH1. Intestinal epithelial tissue-specific transgenic expression of *H19* in mice decreased the levels of ATOH1 protein and led to defects in Paneth cells, goblet cells, tuft cells, and enteroendocrine cells. In contrast, ectopically expressed ATOH1 in primarily cultured intestinal organoids derived from H19-Tg mice rescued the development of secretory lineage cells ex vivo. These findings advance our knowledge about the maturation of secretory lineage cells in the intestinal mucosa and reveal the important role of the *H19*/ATOH1 axis in the regulation of secretory lineage cell function and the epithelial integrity, which in turn influences mucosal injury, inflammation, and gut permeability in various pathological conditions.

Our previous study has shown that the mucosal levels of *H19* in the intestine increase remarkably in mice exposed to septic stress induced by cecal ligation and puncture (CLP) and in patients with sepsis, along with defective secretory lineage cells, whereas *H19* knockout enhances the function of Paneth and goblet cells and protects the gut barrier against CLP in H19^−/−^ mice [35]. The present study further reveals that there were significant differences in cell type-specific gene expression profiles of secretory lineage cells between H19^−/−^ mice and control littermates. *H19*-deficient Paneth and goblet cells in the small intestine of H19^−/−^ mice exhibited increased expression levels of many genes involved in various cellular processes including macromolecule metabolism, biosynthetic process, gene expression, and RNA metabolism. Conversely, locally increasing the levels of *H19* in the intestinal epithelium of H19-Tg mice caused defects in secretory lineage cells, as evidenced by a significant decrease in the numbers of Paneth cells, goblet cells, tuft cells, and enteroendocrine cells, although increased levels of *H19* failed to affect mucosal growth in the small intestine. Importantly, transgenic expression of *H19* in the intestinal epithelium also disrupts gut barrier function, since increasing the levels of tissue *H19* not only decreased levels of tight junction proteins but also increased basal level of gut permeability in H19-Tg mice. These findings clearly demonstrate the essential role of *H19* in regulating development and function of the secretory lineage cells in the intestinal epithelium and subsequent gut barrier function.

The results presented here also indicate that *H19* represses the development of secretory lineage cells at least partially by inhibiting ATOH1 expression. ATOH1 is a master transcription factor in prosecretory cells by activating the expression of other transcription factors that induce differentiation of specific secretory cells [3,47,48]. In this regard, SOX9 and SPDEF are necessary for differentiation of Paneth cells and goblet cells, respectively, and TCF4 controls the balance between Paneth and goblet cells [8,43,49]. POU2F3 induces tuft cell maturation, while neuroG3 is involved in the generation of enteroendocrine cells [3,50]. Activation of ATOH1 by overexpression of its transgene or ablation of its natural repressor HES1 causes excessive differentiation of prosecretory cells into all secretory cells [51,52]. In contrast, targeted deletion of the *Atoh1* gene leads to depletion of whole secretory lineages in the intestinal epithelium [53]. Although transgenic expression of *H19* also decreased the levels of SOX9, SPDEF, TCF4, and POU2F3 in the intestinal mucosa, defects in the secretory lineage cells in H19-Tg mice may result primarily from reduction in the levels of tissue ATOH1. Ectopically expressed ATOH1 in the *H19*-enriched intestinal organoids derived from H19-Tg mice restored development of all secretory cell lineages in an ex vivo model. Since neither increased *H19* in H19-Tg mice nor ATOH1 overexpression in intestinal organoids altered growth of the intestinal mucosa, the regulation of secretory lineage cells by *H19* via ATOH1 results from the effect of *H19* on cell differentiation rather than on ISC proliferation and mucosal turnover.

Our results further show that *H19* regulates ATOH1 expression in the intestinal epithelium through control of ATOH1 translation without effect on its gene transcription. Increased *H19* did not affect the levels of *Atoh1* mRNA in vivo nor in vitro but it inhibited the rate of newly synthesized ATOH1 protein in cultured IECs. This inhibitory effect of *H19* on ATOH1 expression is specific, since *H19* overexpression failed to alter the levels of HES1 protein. *H19* has multiple cellular functions by acting on specific targets via different molecular mechanisms, particularly through the *H19*/miRNA/mRNA-competing endogenous RNA network, where *H19* functions as a mRNA sponge [21,54,55]. *H19* modulates skeletal muscle metabolism, differentiation and regeneration by acting as an upstream regulator of the miRNA let-7 that inhibits expression of critical metabolic genes such as *Insr* and *Lpl* [56,57]. *H19* also functions as an RNA decoy for miR-34b and let-7, two microRNAs involved in controlling the expression of p53 and other apoptosis-associated proteins [32,36]. *H19* controls expression of the imprinted network genes by recruiting repressive histone markers to their differentially methylated regions and it inhibits placental growth through the processing of the embedded miR-675 [37,58]. *H19* interacts with the RBP HuR that decreases miR-675 levels by inhibiting its processing from *H19* [37]. Our previous study reveals that *H19* represses expression of intercellular junction proteins ZO-1 and E-cadherin and impairs the epithelial barrier function in cultured IECs by acting as a regulatable reservoir of miR-675 [21]. However, the exact mechanism by which *H19* inhibits ATOH1 translation remains unknown and should be fully investigated in a future study.

In summary, our results from studies of mice, intestinal organoids, and IECs indicate that by down-regulating ATOH1 translation, *H19* inhibits development and function of secretory lineage cells in the small intestinal epithelium. Tissue-specific transgenic expression of *H19* in mice decreased the levels of differentiation-associated transcription factors including ATOH1, which was associated with defects in the secretory lineage cells in the mucosa. Importantly, ectopically expressed ATOH1, by transfection with its expression vector, restored development of all secretory cells in the *H19*-enriched organoids derived from H19-Tg mice. Moreover, *H19* inhibits ATOH1 expression at the translation level, and this regulation of ATOH1 by *H19* is critical for maintaining secretory cell development and function. The present study strongly supports a novel model whereby rapidly altered levels of mucosal *H19* in stressful environments functionally regulate ATOH1 expression to affect maturation and function of secretory lineage cells, thus contributing to the epithelium-host defense and effectiveness of the gut barrier function. However, there are only limited results showing the molecular mechanism underlying the control of ATOH1 translation by *H19* in this study, and the exact role of altered *H19* in the pathogenesis of IBD and sepsis-induced gut mucosal disruption remains largely unknown. Since the levels of tissue *H19* increase dramatically in patients with critical illnesses such as sepsis and gut mucosal inflammation [35,36], these findings provide a better understanding of control of the secretory cell function in pathological conditions and identify the *H19*/ATOH1 axis as a potential therapeutic target for interventions to preserve the intestinal epithelial integrity and gut barrier function in the clinical setting.

## 4. Materials and Methods

### 4.1. Animal Studies

All animal experiments were performed in accordance with NIH guidelines and were approved by the Institutional Animal Care and Use Committee of University Maryland School of Medicine and Baltimore VA hospital. Wild-type C57BL/6J mice (male and female, 6 to 9 weeks old) were purchased from The Jackson Laboratory (Bar Harbor, ME, USA). H19^−/−^ mice were kindly provided by Dr. Karl Pfeifer, NIH [39], and they carried a 1-kb deletion of *H19* exon 1 that removes almost the entire exon 1, including the region encoding miR-675, and reduces levels of even the partial *H19* transcript by >100-fold globally [35,39,59].

H19-Tg mice were generated by using A33-promoter and following the strategy as we used in the generation of miR-195, miR-222, and vtRNA1-1 transgenic mice [42,60,61]. The 2.2-kb fragment flanking the mouse *H19* locus (including exon1) was cloned into the pIRES-AcGFP1-Nuc vector. Then, the final *H19* expression vector, A33-*H19*/GFP, was injected into fertilized murine eggs [61,62]. Harvard University’s Genome Modification Facility established transgenic founder mice on pure C57BL/6J background by pronuclear injection. The first generation of recombinant mice with H19/GFP bicistronic RNA was identified by PCR genotyping of DNA extracted from tail clippings. Two founder mice were further characterized to subsequently establish transgenic colonies. To include proper littermate control mice, we crossed male Tg mice with wild-type female mice to generate wild-type and *H19* transgene-positive mice. To exclude the possible effect of the A33-GFP transgene, A33-GFP mice were used as pseudo-transgene controls. All H19^−/−^, H19-Tg, and control littermate mice were housed in a specific pathogen-free breeding barrier and cared for by trained technicians and veterinarians.

Animals were deprived of food but allowed free access to tap water for 24 h before experiments. To examine gut mucosal growth in transgenic and control mice, BrdU was incorporated into intestinal mucosa via intraperitoneal (i.p.) injection of 2 mg BrdU (Sigma, St. Louis, MO, USA) diluted in phosphate-buffered saline. Two portions of the small intestine 0.5 cm distal to the ligament of Trietz were removed. One segment was used for histological examination, while the other segment was reserved for extraction of protein and RNA. The mucosa was scraped with a sterile cell scraper for various measurements as described previously [24,43]. Representative results from two independent founder mice were reported and compared with data obtained from control littermate mice.

### 4.2. Cell and Intestinal Organoid Culture

Human colorectal carcinoma Caco-2 cells originally sourced from the American Type Culture Collection (Manassas, VA, USA) were maintained under standard culture conditions [20,24]. The basal medium and fetal bovine serum were purchased from Invitrogen (Carlsbad, CA, USA), and biochemical reagents were purchased from Sigma (St. Louis, MO, USA). Isolation and culture of primary enterocytes were conducted following the protocol provided by STEMCELL Technologies (Cambridge, MA, USA) with minor modifications as described previously [35,45]. To briefly summarize, primary intestinal crypts were first isolated from murine small intestinal tissue segments. Then, isolated intestinal crypt fractions were assessed, suspended in Matrigel (Corning, Tewksbury, MA, USA), and cultured in mouse IntestiCult^™^ organoid growth medium (STEMCELL technology, Vancouver, BC, Canada). The growth of enteroids was examined under phase-contrast microscopy as described.

### 4.3. Plasmid Construction

An expression vector containing a full-length of cDNA encoding ATOH1 under the control of pCMV promoter was purchased from Origene (Rockville, MD, USA) and used to increase the ATOH1 levels in intestinal organoids as described previously [21,46]. Transient transfections were performed using the Lipofectamine reagent following the manufacturer’s recommendations (Invitrogen). Forty-eight hours after transfection, organoids were harvested for analysis.

### 4.4. Isolation of Intestinal Epithelial Cells and scRNA-Seq Analysis

The small intestinal mucosal tissues harvested from littermate and H19 knockout mice were weighed, washed in cold D-PBS and sectioned with a scalpel, as described previously [23]. The washed mucosal tissue segments were incubated in chelation medium (20 mM EDTA-DPBS) at 37 °C for 90 min with gentle agitation. Then, villus-rich supernatant was collected and filtered through a 40 μm filter. The pellet is further dissociated with TrypLE for 10 min at 37 °C (crypt-rich cells) and later combined with villus-rich fragment. The epithelial single-cell suspension was washed in DPBS with 1% BSA and passed through 40 μm filters. The purity of epithelial populations was confirmed by flow-cytometry analysis before proceeding to scRNA-sequencing. Once viability and EPCAM purity were confirmed, these high-quality intestinal epithelial cells were directly loaded for droplet-based scRNA-sequencing according to the manufacturer’s protocol for the Chromium Single Cell Platform (10X Genomics, 3’V2) to obtain ~10,000 cells per reaction. The single-cell sequencing library preparation was carried out according to the manufacturer’s protocol. Using the Cell Ranger software v.9.0.1 and mm10-2020-A transcriptome as a reference, 10× Genomics scRNA-seq gene expression raw sequencing data were processed. Following previous established methods [40,41], the dimensionality reduction, Leiden clustering, differential abundance analysis, cell-type composition analysis, gene enrichment analysis, and mitochondrion Gene Ontology Term (GO: 0005739) were performed with these samples. The threshold used for identifying differentially expressed genes is *p*-value < 0.05 and log2(Fold-change) > 0.25.

### 4.5. Histology and Immunofluorescence Staining1

Dissected and opened intestines were mounted onto a solid surface and fixed in formalin and paraffin. Sections of 5 μm thickness were stained with H&E for general histology. As described previously [46], the overall length of villus and crypts of each intestinal section was measured by using a calibrated micrometer eyepiece. Immunofluorescence staining was performed according to established protocols [11,12]. For experiments analyzing murine mucosal tissue samples, more than five 5 μm-thick slides (5 μm thickness section) per tissue sample were prepared for immunofluorescence staining. For studies utilizing cultured intestinal organoids, slides were fixed in 3.7% formaldehyde in PBS and rehydrated. All slides were incubated overnight with a primary antibody in blocking buffer recognizing lysozyme, mucin 2, DCLK1, chromogranin A, or E-cadherin, then incubated with secondary antibody conjugated with Alexa Fluor-594 (Cat No. A32754, Molecular Probes, Eugene, OR, USA) for 2 h at room temperature. After rinsing 3 times, the slides were incubated with DAPI (Cat No. D1306, Molecular Probes, Thermo Fisher Scientific, Eugene, OR, USA) to stain cell nuclei at a concentration of 1 μM for 10 min. Finally, the tissue slides were washed, mounted, and imaged through a Zeiss confocal microscope (model LSM700, Carl Zeiss Microscopy GmbH, Jena, Germany). To ensure unbiased results, the slides were examined while coded, and they were decoded only after data collection was completed. Images were processed using Photoshop software version 25.0 (Adobe, San Jose, CA, USA).

### 4.6. Measurement of Gut Permeability

FITC-conjugated dextran diluted in sterile 1x phosphate-buffered saline (Cat No. FD4, Sigma; 4KD; 600 mg/kg) was administered to mice via oral gavage as described [42]. After 4 h, blood was collected via cardiac puncture. The serum concentration of the FITC-dextran suspension was examined using a fluorescence plate reader set at an excitation wavelength of 490 nm and an emission wavelength of 530 nm. The concentration of FITC-dextran in sera was determined by comparing the values to the FITC-dextran standard curve.

### 4.7. Reverse Transcription and Q-PCR Analysis

Total RNA was extracted by using RNeasy mini kit (Qiagen, Valencia, CA, USA), and reverse transcription and subsequent PCR amplification reactions were performed as described [23]. After performing RT, quantitative PCR (Q-PCR) analysis was conducted using 7500-Fast Real-Time PCR Systems with specific target primers, probes, and software (Applied Biosystems, Foster City, CA, USA). The levels of *Gapdh* PCR product were measured in RT- quantitative (q) PCR samples to account for potential variations in RNA input. For miRNA studies, miR-675 levels were also quantified by Q-PCR following the Taqman MicroRNA assay. Small nuclear RNA (snRNA) *U6* levels were measured as an endogenous control. All Q-PCR primers were purchased from Thermo Fisher Scientific (Waltham, MA, USA).

### 4.8. Western Immunoblotting and Newly Synthesized Protein Assays

Whole-cell lysates were prepared by using the RIPA buffer (50 mM Tris pH 7.4, 150 mM NaCl) containing 1% sodium dodecyl sulfate (SDS), sonicated, and centrifuged at 4 °C for 15 min [35]. The supernatants were boiled and size-fractionated by SDS-PAGE. After the blots were incubated with primary antibody and then secondary antibodies, immunocomplexes were developed by using chemiluminescence. Relative protein levels were analyzed by using Biorad Chemidoc (Bio-Rad Laboratories, Inc., Hercules, CA, USA) and XRS system equipped with Image lab software (version 4.1), and the values were normalized with internal loading control HSC70.

In addition, new protein synthesis was measured by Click-iT Protein Analysis Detection Kit (Life technologies, Grand Island, NY, USA) following the manufacturer’s manual. First, Caco-2 cells were incubated with methionine-free medium. Then, to label nascent protein synthesis, cells were exposed to and metabolically incorporated azide-modified amino acid L-azidohomoalanine (AHA). Next, the cell lysates were combined with the reaction buffer and incubated for 20 min. Following incubation, paramagnetic Streptavidin-conjugated Dynabeads (Invitrogen, Thermo Fisher Scientific, Waltham, MA, USA) were used to pull down biotin-alkyne/azide-modified protein complexes. Finally, the precipitated samples were resolubilized in 10% SDS-PAGE and analyzed by Western immunoblotting analysis using antibodies against ATOH1 or HSC70 as a loading control.

### 4.9. Chemicals and Antibodies

Biochemicals were purchased from Sigma (St. Louis, MO, USA), and culture medium and fetal bovine serum were from Invitrogen (Carlsbad, CA, USA). Antibodies recognizing lysozyme (sc-518012), DCLK1 (Cat No. ab31704), mucin 2 (Cat No. ab272692), chromogranin A (Cat No. PA5-77917), ZO-1 (Cat No. 5406S), occludin (Cat No. 91131S), claudin-1 (Cat No. 37-4900), E-cadherin (Cat No. 610182), ATOH1 (Cat No. PA5-29392), SOX9 (Cat No. 82630S), SPDEF (Cat No. 11467-1-AP), TCF4 (Cat No. 2569T), POU2F3 (Cat No. HPA019652), neuroG3 (Cat No. 703206), HES1 (Cat No. 11988S), PCNA (Cat No. 2586S), HuR (Cat No. SC5261), CUGBP1 (sc-5261), and HSC70 (MABE1120) were obtained from Cell Signaling Technology (Danvers, MA, USA), Invitrogen, Santa Cruz Biotechnology, BD Biosciences (Sparks, MD, USA), Proteintech (Rosemont, IL, USA), and Atlas Antibodies (Stockholm, Sweden). The secondary antibody conjugated to horseradish peroxidase was purchased from Sigma. All antibodies utilized in this study were validated for species specificity. Antibody dilutions used for Western immunoblotting analysis were 1:800 or 1000 (first Ab) and 1:2000 (second AB), respectively, whereas antibody dilutions for immunostaining were 1:200 (first) and 1:2000 (second).

### 4.10. Statistical Analysis

All data values were presented as the means ± SEM, derived from five animals or three separate experiments. When applicable, an unpaired, two-tailed Student’s *t*-test was used to determine statistical significance *p* < 0.05. For multiple group comparisons, one-way ANOVA was utilized with Tukey’s post hoc test [63]. Non-parametric rank comparisons were evaluated using the Kruskal–Wallis test. All statistical analyses were conducted in GraphPad Prism 9.0 (San Diego, CA, USA). For non-parametric analysis rank comparison, the Kruskal–Wallis test was conducted.

## Data Availability

All data are available upon request due to the process of data deposition.
